# Multifaceted Interventions Based on Education and Recognition to Enhance Nurses' Compliance With Standard Precautions: A Quasi‐Experimental Design

**DOI:** 10.1111/nhs.70334

**Published:** 2026-04-12

**Authors:** Menevse Yildirim, Seyda Seren Intepeler, Simon Ching Lam

**Affiliations:** ^1^ Department of Nursing Management, Faculty of Nursing Dokuz Eylul University Izmir Turkey; ^2^ School of Nursing Tung Wah College Hong Kong SAR China

**Keywords:** education, infection control, nurses, occupational health, recognition, standard precautions, universal precautions

## Abstract

This study examines the effect of multifaceted interventions based on education (1) and recognition (2) of compliance with standard precautions (SPs). A quasi‐experimental research design with a one‐group pre‐test and post‐test was used. The purposive sample included 71 nurses working in different specialty units at a large hospital in Turkey. The researchers developed multifaceted interventions informed by existing literature, data from the hospital, and insights from frontline experts, including nurses, ward managers, infection control officers, and health and safety officers. Initially, modifications were implemented in the physical environment of the clinics, and material deficiencies were addressed. In the subsequent phase, nurses engaged in an educational session focused on SPs. The nurses completed the Compliance with Standard Precaution Scale (CSPS) three times, including baseline (before the intervention, T0), third month after the intervention (T1), and sixth month after the intervention (T2). Nurses' median compliance with SPs was significantly higher at T2 (15.8 ± 3.42) than at T0 (13.7 ± 3.16, *p* < 0.05). The multifaceted interventions comprising structured education and recognition enhanced the compliance with SPs of clinical nurses.

## Key Points

- This study outlines exemplary interventions to improve nurses' compliance with SPs.
- The interventions related to providing education and recognition improved nurses' compliance scores. To enhance compliance with SPs, a minimum duration of 6 months is needed.
- Policymakers and senior managers should invest in enhancing nurses' compliance with SPs. It is important to effectively organize the work environment and address a lack of training and resources.

## 1 Introduction

Healthcare workers (HCWs) accept SPs as a protective measure to prevent exposure to injuries, accidents, and diseases that they may encounter during their professional care (Moshksar et al. 2023). Compliance of HCWs with SPs varies globally and ranges between 12% and 76% (Haile et al. 2017; Cruz et al. 2024). Various studies have determined that HCWs either select infection control precautions inadequately, apply them insufficiently, or fail to apply them altogether (Moshksar et al. 2023; Lam 2011; Goudra et al. 2014). In the literature, rates of hand hygiene compliance as a necessary precaution have been reported to range from 14.9% and 28.5% (Engdaw et al. 2019; Harun et al. 2023), while rates of glove use range between 39.6% and 77.5% (Goudra et al. 2014; Holla et al. 2014). A study found that 79.8% of HCWs recapped needles after injection, while 15.2% failed to wear an apron/gown when required (Angeloni et al. 2024). Protective masks are worn by only 45% of HCWs in situations presenting possible exposure to blood and body fluids (Diniz et al. 2023), while 70.9% of them cover wounds with waterproof dressing before patient contact (Edward et al. 2024). Although all of these protective measures are correct, the rates indicate that many HCWs do not follow them (Angeloni et al. 2024). Various interventional studies have been conducted to improve nurses' compliance with SPs; utilizing numerous strategies such as training, monitoring, and demonstrations (Tromp et al. 2012; Waramlah and Huda 2019; Nmadu et al. 2016; Mukthar et al. 2017; Gomarverdi et al. 2019; Angeloni et al. 2024). Notably, multifaceted approaches are more effective than single strategies in producing favorable outcomes (Diniz et al. 2023; Tromp et al. 2012). Despite these promising results, some studies lacked clarity regarding the time interval between pre‐ and post‐tests (Tromp et al. 2012; Waramlah and Huda 2019), which makes it difficult to assess the sustainability of the interventions. Study durations varied: while some measured short‐term effects (ranging from 15 days to 1.5 months) (Gomarverdi et al. 2019; Angeloni et al. 2024), others examined outcomes up to 6 months post‐intervention (Nmadu et al. 2016; Mukthar et al. 2017). Given that behavior change typically takes an average of 66 days, ranging from 18 to 254 days (Lally et al. 2010), this study aims to assess both short‐ and long‐term effects, offering a more comprehensive view of intervention effectiveness.

While current findings provide valuable insights, further interventional research is still needed, particularly those exploring structured education and recognition programs, beyond changes in workplace organization, to identify the most effective strategies for ensuring compliance (Waramlah and Huda 2019; Cruz et al. 2024).

### 1.1 Theoretical Framework

Bandura's (1986) Social Cognitive Theory (SCT) provides a comprehensive framework for understanding how individuals acquire and modify behaviors through interactions with their environment (Bandura 1986). SCT emphasizes the importance of self‐efficacy, observational learning, and the dynamic interactions between individuals and their environment in shaping behavior, and it is widely used to explain and predict health behaviors (Waramlah and Huda 2019; Mukthar et al. 2017). To develop effective interventions for compliance with SPs, it is essential to understand the interactions between individual, behavioral, and environmental factors. The core components of SCT, therefore, provide a solid foundation for designing interventions aimed at improving compliance with SPs (Bandura 1986; Schunk and DiBenedetto 2020; Manjarres‐Posada et al. 2020), and their application can be seen in the following key areas.

### 1.2 Core Components of SCT and Their Implementation

- *Self‐efficacy*: Refers to individuals' assessment of their abilities. For example, designing educational sessions can increase nurses' knowledge and confidence in adhering to SPs.
- *Outcome expectations*: Factors that motivate individuals to perform a behavior based on their belief in the expected outcomes. In this case, outcome expectations can be shaped by emphasizing the personal and institutional benefits of SP implementation during training and offering participation certificates.
- *Reciprocal determinism*: This concept highlights the interaction between individuals and their environment in shaping behavior. For example, collaborating with hospital management to improve physical conditions, such as reorganizing waste disposal areas and isolation rooms, may impact nurses' behavior.
- *Self‐regulation*: The ability to control and regulate one's behavior. This can be facilitated by conducting regular clinical visits after training sessions to address any questions or concerns nurses may have.
- *Observational learning*: This component emphasizes the influence of observation and interpersonal interactions in learning or modifying behavior. For instance, placing reminder posters around the clinics can help improve compliance with SPs.
- *Facilitation*: This refers to providing new structures or resources that make it easier for individuals to change their behavior. Collaborating with hospital management to address material needs and adjusting training schedules according to nurses' shifts are examples of facilitating changes in behavior (Bandura 1986; Schunk and DiBenedetto 2020; Manjarres‐Posada et al. 2020).

In Turkey, compliance with SPs is assessed by analyzing one or several parameters, such as infection control, contagious diseases, and hand hygiene. Only a few studies have investigated this subject (Akyıl and Uzun 2007; Mankan and Kasıkcı 2015). Our preliminary research revealed that the physical layout of the hospital environment significantly influences nurses' behaviors (Samur and Seren Intepeler 2019).

This study, therefore, aims to examine the effect of multifaceted interventions—focused on education and recognition—on nurses' compliance with SPs.

## 2 Materials and Methods

### 2.1 Design

In studies aiming to develop interventions for standard precautions (SPs), one‐group pre‐test–post‐test designs, either with or without a control group, are commonly employed (Waramlah and Huda 2019). This study adopted a quasi‐experimental one‐group pre‐test–post‐test design without a control group. The study design and reporting were guided by the Transparent Reporting of Evaluations with Nonrandomized Designs (TREND) statement (Des Jarlais et al. 2004).

### 2.2 Participants

This research was conducted between August 2018 and December 2019 in the wards of a large Education and Research Hospital in Izmir. This hospital has a bed capacity of 1140 and employs 761 nurses. The primary reason for conducting this study at the institution is the Health Care Services Manager's emphasis on the health of HCWs and their commitment to improving the working environment within the institution. The researchers selected the wards based on the frequency of reported accidents and injuries. In this context, “accident and injury notification forms” submitted to the hospital's Infection Control Unit were reviewed. Among the 309 reported injuries, 120 involved sharp‐edged injuries or contamination with blood or body fluids affecting nurses or intern nurses. After classifying the data by department, the five wards with the highest number of injuries were identified: general internal medicine, gastroenterology, endocrinology, anesthesia, intensive care, and cardiology. These wards number of nurses and hours of operation. The general characteristics of these wards are summarized in Table 1.

**TABLE 1:** General characteristics of the clinics.

	Ward 1	Ward 2	Ward 3	Ward 4^a^	Ward 5
Discipline	Gastrointestinal (esophagus, stomach, small and large intestines) disorders, liver, gallbladder, and bile ducts, digestive functions disorders of the pancreas.	Internal organ disorders such as hypertension, lower and upper respiratory tract diseases, diabetes, thyroid, kidney, and intestinal diseases.	Heart and cardiovascular diseases such as heart attack, heart rhythm disorders, and heart failure.	Diagnosis, treatment, and follow‐up of patients who need to be supported by a respiratory device or whose condition is critical before, during, and after surgery, and needs intensive care.	Diseases of areas such as joints, soft tissues, and hereditary connective tissue, as well as disorders of the endocrine glands.
Number of beds	25	29	25	31	29
Number of nurses (Total)	15	13	12	45	12
Number of nurses working during the day	5	5	5	18	5
Number of nurses working at night	2	2	2	12	2
Shift weekdays	8 h (08.00–16.00) and 16 h (16.00–08.00)	8 h (08.00–16.00) and 16 h (16.00–08.00)	8 h (08.00–16.00) and 16 h (16.00–08.00)	8 h (08.00–16.00) and 16 h (16.00–08.00)	8 h (08.00–16.00) and 16 h (16.00–08.00)
Shift weekends	24 h (08.00–08.00)	24 h (08.00–08.00)	24 h (08.00–08.00)	24 h (08.00–08.00)	24 h (08.00–08.00)
Number of accident and injury reports	17	18	11	12	15

^a^ This ward contains four separate, nested sub‐wards similar to the other ward's size.

Following the identification of the specific wards, the study sample consisted of all nurses working in these wards. Using purposive sampling, all nurses who volunteered to participate in the study were included. Participants who declined to participate, left the ward/or institution during the study, were on legal leave (e.g., maternity leave), or had missing repeated test data were excluded from the study. The researcher visited all clinics, provided the nurses with information about the purpose, scope, and methodology of the study, and personally invited them to participate.

### 2.3 Multifaceted Interventions

#### 2.3.1 Preliminary Phase

Initiatives were identified and implemented within a structured framework through qualitative interviews with nurses at various levels, including charge nurses, novice nurses, and experienced nurses, in the designated wards. These interviews revealed that physical inadequacies in the wards contributed to various accidents and injuries among nurses, including infectious diseases, sharp object injuries, dermatitis, and latex allergies (Samur and Seren Intepeler 2019). Furthermore, the findings indicate a significant gap in nurses' knowledge of SPs to prevent workplace accidents, highlighting the urgent need for training (Samur and Seren Intepeler 2019). In response to these findings, researchers scheduled appointments with charge nurses and organized a meeting that included participating nurses. This collaborative effort aimed to address the identified issues and develop actionable strategies for improvement. During the meeting, nurses primarily requested the implementation of ward‐specific interventions to address physical conditions and material shortages. They also sought information to tackle employee health and safety concerns related to SPs. Consequently, the study was structured around two primary initiatives: (I) the “recognition” and (II) the “educational session” focused on SPs. The preliminary phase for these interventions, which included designing the identified ward‐specific needs, securing funding, procuring materials, developing the educational content, creating reminders, and other related tasks, was completed over 9 months.

#### 2.3.2 An Implementation Phase

##### 2.3.2.1 Recognition

**FIGURE 1:**
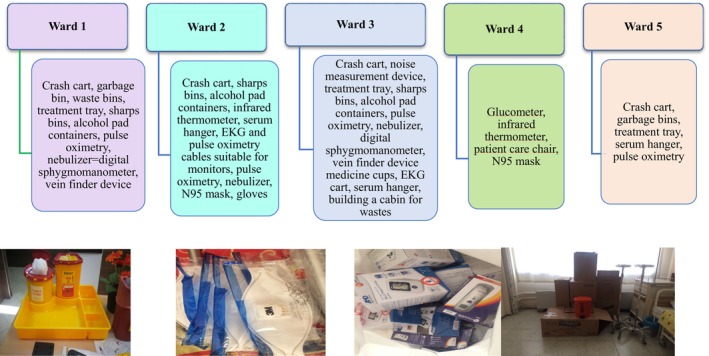
List of ward‐specific material requirements.

The researchers visited each ward on separate days, as scheduled by the charge nurses, to identify ward‐specific needs. Participation in these visits was open to any available and willing nurses. During the visits, the physical conditions of each ward were evaluated for risks of accidents and injuries, material shortages were assessed, and potential improvements for employee health and safety were identified. The recognized needs were subsequently communicated to the upper management, which reviewed these requirements and defined the scope of the necessary changes. The list of defined needs for each ward, which are tailored to their specific requirements, is presented in Figure 1.

The research addressed the specific needs of each ward and completed the procurement processes. The researchers then re‐engaged with the upper management and charge nurses to finalize a collaboration protocol. Subsequently, materials were transferred from the researchers' affiliated faculty to the hospital. Following the transfer, these materials were delivered to all wards on the same day specified by the charge nurses and were placed in designated locations. The researchers maintained ongoing communication and coordination with the hospital support staff and charge nurses throughout this process. In addition to fulfilling material requirements, the following improvements were implemented with the support of the hospital management's human resources and technical expertise.

Ward 2: Isolation rooms were reorganized by removing excess items, such as chairs, hangers, and other unnecessary furnishings, to enhance hygiene and safety.

Ward 3: The waste collection area was overhauled, including the construction of a covered waste bin to enhance sanitation.

##### 2.3.2.2 Education

###### 2.3.2.2.1 Construction of the Educational Content

**FIGURE 2:**
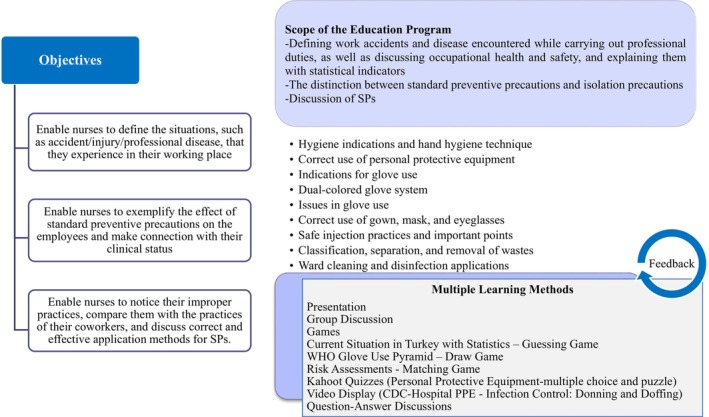
Overview of training program goals, scope, and learning method.

The researchers developed the content of the education focused on the health and safety of HCWs using the Injury Assessment Form (refer to outcomes), data from qualitative interviews (Samur and Seren Intepeler 2019), guidelines from the Centers for Disease Control and Prevention (CDC) and the World Health Organization (WHO) (CDC 1988, 2011; WHO 2007, 2009a, 2009b), and pertinent literature (Waramlah and Huda 2019; Burton 2010; UK. National Clinical Guideline Centre 2012). The educational content was revised based on recommendations from nurses in the “Employee Health and Safety Unit” (two specialist nurses) and the “Infection Control Unit” (two specialist nurses and one nurse) to ensure the validity of its content. The final version of the educational content was developed with contributions from field experts (one professor and one researcher) and input from nurse managers and charge nurses. Figure 2 illustrates the education's objectives, scope, and learning methods.

After the scope of the educational program was finalized, a meeting was convened with the hospital management and charge nurses. During this meeting, the necessary resources for the training (e.g., presentation rooms, computers, and pointers) were identified, and the upper management arranged for their procurement.

###### 2.3.2.2.2 Educational Sessions

The educational sessions were scheduled to occur in either the meeting rooms of the wards or the hospital's conference room, depending on availability. The scheduling of volunteer nurses for the education was organized under the direction of the charge nurses, with each volunteer participating in a single 2 h session. Sessions were scheduled during lunchtime (12:00–14:00) and around shift changes (17:00–19:00) to ensure that all nurses could attend an education despite their shifts. For those who could not attend the initial sessions, a new plan was developed to accommodate their schedules. A total of 24 sessions were conducted by the researcher, with attendance ranging from 3 nurses to 8 nurses per session. Certificates of participation were awarded to the nurses upon completion. Informative brochures regarding SPs, along with pens, notepads, and food, were provided to facilitate adherence to the sessions. Feedback from the nurses is summarized in Table 2.

**TABLE 2:** Nurses' feedback regarding the educational program (*n* = 83).

Items	1		2		3		4		5		Mean	Standard deviation
	*n*	%	*n*	%	*n*	%	*n*	%	*n*	%		
1. The program will increase my compliance with standard preventive precautions.	1	1.2	2	2.4	0	0	35	42.2	45	54.2	4.46	0.738
2. The program will be useful to generate an employee health and safety culture.	1	1.2	2	2.4	0	0	34	41.0	46	55.4	4.47	0.738
**3. I was satisfied with the program score in general**.	**2**	**2.4**	**1**	**1.2**	**2**	**2.4**	**17**	**20.5**	**61**	**73.5**	**4.61**	**0.809**
4. Time for the themes was sufficient.	1	1.2	2	2.4	8	9.6	25	30.1	47	56.6	4.39	0.853
5. The themes were clearly and explicitly stated.	2	2.4	1	1.2	0	0	24	28.9	56	67.5	4.58	0.783
6. I understood the information provided in the session.	2	2.4	1	1.2	2	2.4	22	26.5	56	67.5	4.55	0.815
7. I am planning to use the information from the session.	2	2.4	1	1.2	0	0	37	44.6	43	51.8	4.42	0.783
8. I found the information given in the session useful.	2	2.4	2	2.4	1	1.2	26	31.3	52	62.7	4.49	0.846
9. Learning activities made learning easier.	2	2.4	1	1.2	1	1.2	30	36.1	49	59.0	4.48	0.802

*Note:* 1: Definitely disagree; 2: Disagree; 3: Indefinite; 4: Agree; 5: Definitely agree. The bold values indicate the item with the highest mean score for emphasis and do not represent statistical significance.

**FIGURE 3:**
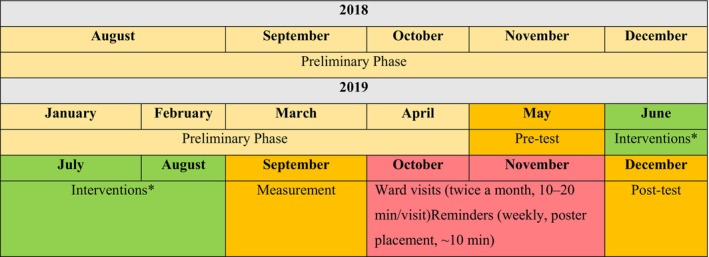
General pattern of implementation of the interventions. *Initially, the clinical materials were distributed to all wards on the same day, and the training program was conducted while the staff utilized these materials. Each training session lasted approximately 2 h, and the overall training period spanned 3 months.

The interventions across all wards were conducted over 3 months and facilitated by the researchers. During the subsequent 3–6 months following the educational session, the researchers visited the wards every 15 days to address and resolve inquiries from nurses regarding SPs. In addition, seven posters were consistently displayed in locations recommended by the nurses. In the fourth month, coasters were distributed to nurses as gifts and interim reminders. The general pattern of implementation of the initiatives is shown in Figure 3.

### 2.4 Objectives

This study aimed to examine the effects of multifaceted interventions, which included the education and recognition of compliance with SPs.

The null hypothesis of this study posits no significant difference in compliance scores regarding SPs before and after the implementation of multifaceted interventions.

### 2.5 Outcomes

The researchers visited the wards before, during, and after the intervention to distribute the data collection forms (1, 2, and 3) and collected them within 3–5 days.

#### 2.5.1 Primary Study Outcome

##### 2.5.1.1 CSPS (1)

The CSPS is an instrument designed to measure employees' self‐reported compliance with SPs in clinical settings. The scale was developed by Lam (2011) based on the guidelines established by the CDC and the WHO. The four‐point adjectival scale (never, seldom, sometimes, and always) consists of 20 items. Items 2, 4, 6, and 15 are negatively worded; therefore, their scores are reversed before calculation. The total scale score ranges from 1 to 20, with higher scores indicating better compliance with SPs (Lam 2014). The content validity index of the original scale is 0.90, the Cronbach's alpha coefficient is 0.73, and the intraclass correlation coefficient is 0.79 (Lam 2011). The CSPS was adapted and psychometrically tested using the Rasch measurement method for application in Turkish. Psycholinguistic and psychometric evaluations indicated that the Turkish version of the CSPS (CSPS‐T) is suitable for Turkish nurses and represents a unidimensional, homogeneous construct. The Cronbach's alpha (0.71), corrected item‐total correlations, and intraclass correlation coefficients (0.84) were all high and deemed acceptable (Samur et al. 2020).

##### 2.5.1.2 Introductory Characteristic Question Form (2)

The researchers designed the form, which included questions regarding age, gender, years of professional experience, and weekly working hours.

##### 2.5.1.3 Assessment Form for Educational Session (3)

Considering the study of Shen et al. (2018), the assessment items of the educational session were reorganized within the context of an education initiative focused on the SPs. After the educational session, nurses assessed the training using a five‐point Likert scale. The response options were: (1) definitely disagree; (2) disagree; (3) neutral; (4) agree; (5) definitely agree. The Cronbach's alpha reliability coefficient was 0.97.

### 2.6 Sample Size Estimation

A priori power analysis was conducted under conventional conditions. The results indicated that a sample size of at least 43 nurses was necessary to detect a medium effect size (effect size *f* = 0.25), with a Type I error of 0.05 and a Type II error of 0.05 (95% power). Consequently, including 97 nurses from the departments was considered sufficient for the target sample. Volunteers from Ward 1 (13 nurses), Ward 2 (8 nurses), Ward 3 (10 nurses), Ward 4 (32 nurses), and Ward 5 (8 nurses) participated in and completed all three assessments. The study concluded with a total of 71 nurses.

### 2.7 Statistical Methods

The data obtained in the research were analyzed using the Statistical Package for the Social Sciences (SPSS) version 25.0. Descriptive statistical methods (number, percentage, median, mean, and standard deviation) were used to evaluate the data, and Cronbach's alpha reliability coefficient was employed to assess the internal consistency of the scale. Normal distribution of the data was determined by examining whether the skewness and kurtosis values fell within the range of ±3 (Kim 2013). In our study, nurses' compliance with SPs was measured repeatedly at different time points, and these data did not follow a normal distribution. Therefore, non‐parametric statistical methods were applied for analysis. The Friedman test was conducted to detect differences in compliance with SPs among T0, T1, and T2. The Bonferroni multiple comparison tests were performed to identify distinct groups in the data analyzed using the Friedman test, aiming to control the Type I error rate due to multiple comparisons. Only the cases with complete data, that is, no missing values for any of the variables, were included in the analysis. The *p* value less than 0.05 is set as significant.

### 2.8 Ethical Approval and Informed Consent

To conduct the research, institutional permissions were obtained from the Noninvasive Research Ethics Committee where the researchers were affiliated (Date: 10.03.2016, Decision No: 2016107‐27), from the scale developer (Lam 2011), and from the Education and Research Hospital where the study was conducted. Informed consent was obtained from the nurses before the training. All the information provided by the nurses was kept confidential.

## 3 Results

**FIGURE 4:**
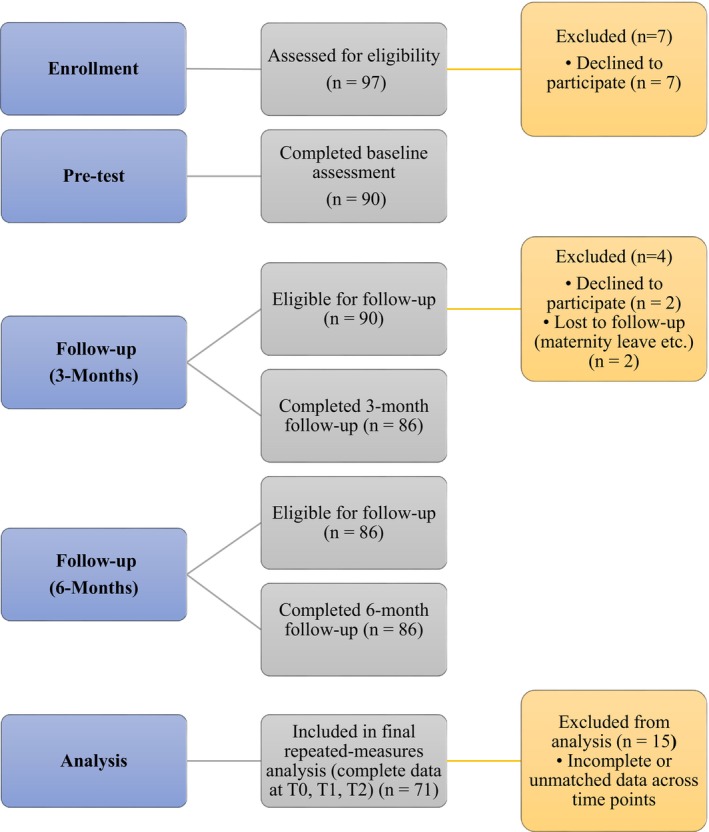
The flow chart depicting sample attrition.

During the pre‐test implementation, 90 nurses out of 97 worked in the selected hospital wards (7 refused to fill out the form, with a response rate of 92.7%). In the third month, 86 nurses (9 refused to fill out the form, with a response rate of 90.5%) out of 95 nurses (2 on maternity leave) filled out the data collection form. In the sixth month, 86 nurses (2 refused to fill the form, a response rate of 97.7%) out of the actively working 88 nurses (2 on maternity leave, 7 left the institution/unit) filled the data collection form. From the pre‐test T0, third‐month T1, and sixth‐month assessments T2, 15 unmatched datasets were not used. In conclusion, analyses were conducted with 71 matching datasets. The flow diagram illustrating the sampling of nurses is shown in Figure 4.

A total of 90.1% of the nurses who participated in the research were female. Among the nurses, 36.6% were in the age group of 20–25 years, with a mean age of 30 ± 6.30. In addition, 54.9% of them held an undergraduate degree. The average length of clinical experience for the nurses was 8.32 ± 0.77 years. The average professional experience among the nurses was 10.90 ± 1.88 years, and the average weekly working hours were 50.70 ± 1.06.

The median total score of the participant nurses who received the CSPS applied as a pre‐test was 14.0 out of a total score of 20 at T0, 15.0 at T1, and 17.0 at T2. Based on the Friedman analysis results, a statistically significant difference was found between the median total scores of the repeated CSPS (*p* < 0.05). We hereby reject the null hypothesis. The Bonferroni post hoc test results revealed significant differences in CSPS median scores across three timeslots, with T2 scores significantly higher than those at T0 and T1 (Table 3).

**TABLE 3:** Mean scores of the CSPS in the pre‐test, in the third and sixth months, and the Friedman test results (*n* = 71).

Scale	Beginning of the program (T0)				The third month (T1)				The sixth month (T2)				*X* ^2^ ^b^	*p* *	Bonferroni
	Min	Max	Median	*X̄* ± SD^a^	Min	Max	Median	*X̄* ± SD	Min	Max	Median	*X̄* ± SD			
CSPS	0.0	20.0	14.0	13.7 ± 3.16	6.0	19.0	15.0	14.5 ± 2.88	2.0	20.0	17.0	15.8 ± 3.42	32.889	0.000*	3 > 1 3 > 2

^a^ *X̄* ± SD: mean and standard deviation.

^b^ $X^{2}$: Friedman analysis.

* *p* < 0.05.

## 4 Discussion

Most studies have reported that structured training programs applied to nurses can enhance their compliance with SPs (Gomarverdi et al. 2019; Cheung et al. 2015; Holmen et al. 2016; Gammon et al. 2008; Bikmoradi et al. 2013; Stock et al. 2016). However, structuring the education program by multiple/active learning methods (e.g., visual–audial materials and web‐based educational tools) rather than a program that only includes direct instruction can offer additional benefits (Nmadu et al. 2016; Gomarverdi et al. 2019; Stock et al. 2016). Similar to this study, an intervention work that utilized multiple learning methods conducted repetitive measurements before and 6 months after the intervention. The mean scores for compliance with SPs increased from 7.4 to 8.4 (Tromp et al. 2012). In another study conducted with registered nurses, the mean scores for compliance with SPs increased from 3.7 to 5.9 by the end of the sixth month using the structured training program (Mukthar et al. 2017). In another study, improvements were observed in nurses' practices of handwashing (mean score = 24.20–28.55), gloving (mean score = 2.03–4.42), gowning (mean score = 26.15–35.07), and masking (mean score = 9.35–13.58). The pre‐test and post‐test scores of the nurses exhibited a significant difference. However, no effect was observed on handling sharp objects (mean score = 6.00–6.35) and waste disposal (mean score = 6.57–6.82) (Adly et al. 2014). Unlike the present study, some studies significantly increased nurses' compliance scores with SPs.

In the study of Gomarverdi et al. (2019), intensive care unit nurses received training on handwashing, using Personal Protective Equipment (PPE), and waste disposal, among other techniques. Nurses' compliance with SPs was assessed 2 and 6 weeks after the intervention. The study found that nurses' compliance with SPs increased from 19.8% to 29.2% to 28.4%. The study was conducted with a smaller sample size in only one unit. In addition, an evidence‐based guide regarding the SPs was provided to the employees, which might have created an environment that facilitated the internalization of SPs for nurses. In the study of Holmen et al. (2016), the hand hygiene compliance rates increased from 34% to 68% within 2 weeks through interventions for hand hygiene (e.g., introducing alcohol‐based hand antiseptics, providing mini pocket antiseptic solutions to employees, and giving reminders). Another study with a significant increase in scores was conducted by Duerink et al. (2006). In the study, washbasins were installed in the wards, observations were conducted, feedback was provided after the observations on performance, and educational strategies were implemented. Therefore, these interventions were found to significantly improve and sustain hand hygiene compliance among nurses (from 46% to 77% in Internal Medicine, and from 22% to 62% in Pediatrics). They also enhanced the correct closure of needle sticks (by 20%), which led to a reduction in unnecessary mask use. Therefore, nurses' compliance may increase further with the implementation of various strategies in the studies. These strategies could include reducing the number of clinics (to two or three), concentrating solely on specific behaviors, conducting observations, and providing feedback on performance. This study was conducted in five wards with varying characteristics, and the sample size of nurses was larger than in other studies. This condition might have decreased the interaction with the nurses and negatively impacted their compliance. Furthermore, the long interval between the tests may have led to a decrease in the effectiveness of the education over time. Additionally, the long interval between the pre‐ and post‐tests may have diminished the sustained impact of the educational intervention. Furthermore, although no test‐related cues or feedback were provided, nurses' prior exposure to the test could have influenced their responses, resulting in pre‐test sensitization. The published studies state that education alone is insufficient to influence compliance with SPs. More successful outcomes can be achieved through corporate‐level interventions that aim to impact an institution's culture. These interventions may include practices such as giving and receiving feedback, making observations, providing adequate materials, and utilizing role models (Gammon et al. 2008; Jansson et al. 2016). Henderson (2001) stated that information is necessary to influence compliant behaviors, but it is inadequate; therefore, enhancing the working environment is considered a significant milestone. Materials regarding certain precautions (e.g., hand hygiene) were provided to the clinic (Creedon 2005; Holmen et al. 2016) in only a few studies. However, meeting clinical needs comprehensively and effectively is a challenge that needs to be addressed at an executive level. In the present study, along with providing education and recognition, offering material support to the wards through a requirement analysis indicates that using various strategies together is crucial to influencing compliance with SPs. Moreover, especially in the second phase of the study, strategies such as seeking nurses' opinions to enhance their work environment, regular communication with mid‐level managers, and conducting ward visits/reminders may have motivated the nurses and boosted their willingness. In the context of the present study, commonly used methods in educational sessions, such as presentations and video screenings, were also employed. In contrast to other studies, this research also incorporated Kahoot, gamification, scenarios, and group discussions. In particular, the use of these tools may enhance nurses' attention, especially among the new generation, and help them stay focused on the subject for longer periods. However, despite these potential benefits, the implementation of educational sessions during lunch breaks or shift changeovers may have inadvertently increased nurses' workload, potentially resulting in stress and fatigue. Moreover, the inability of some nurses to fully disengage from their clinical duties during the sessions might have reduced the overall effectiveness of the training.

In light of these challenges, several limitations should be discussed. Firstly, the absence of a control group is a significant limitation in this study, affecting internal and external validity. Implementing this interventional study in a single hospital made assigning a control group challenging due to practical reasons such as the risk of contamination bias between clinics (Ghorbanmovahhed et al. 2023), limited human and financial resources (Shek and Zhu 2018), and doctoral dissertation time constraints.

Secondly, the study faced a degree of participant attrition across its stages. While some losses were due to uncontrollable external factors such as maternity leave or resignation, others resulted from incomplete forms or missing data across the three measurement points. Only two participants declined to continue without providing specific reasons. Of the 97 nurses initially recruited, approximately 75% completed all three data collection stages. Although attrition may have introduced a risk of selection bias, its potential impact was minimized by analyzing only those cases with complete data sets across all time points, thereby ensuring consistency and comparability in the results.

Lastly, the presence of confounding factors that may influence nurses' behaviors—such as individual factors (e.g., lack of knowledge, forgetfulness, the belief that precautions are unnecessary, willingness, and openness to development), unit‐specific factors (e.g., patient population, team collaboration), and organizational factors (e.g., managerial attitude, hospital environment), constitutes a significant limitation (Kim et al. 2025; Oh and Choi 2019). To minimize the impact of these confounding variables, the researchers conducted preliminary interviews with upper managers, charge nurses, and clinical nurses, implementing interventions within the framework of a comprehensive needs analysis.

## 5 Conclusions

Interventions were found to lead to an increase in the total median scores of repeated assessments. However, 3 months was inadequate to produce a significant difference. Therefore, at least 6 months would be necessary to observe a significant impact. Top managers should provide primary materials for care (N95 masks, gloves, waste bins, sharps bins, etc.) and educational requirements in the workplace to enhance nurses' compliance with SPs.

## 6 Relevance for Clinical Practice

The study outlines effective strategies for enhancing compliance with SPs. Policymakers and senior managers must develop and implement policies that promote nurses' compliance with SPs, as this is critical for effective infection control. These policies should be tailored to address the specific needs of departments. Furthermore, upper and middle management must ensure that nurses are provided with the necessary training, resources, and equipment to perform their duties efficiently. Additionally, a minimum follow‐up period of 6 months should be established to evaluate the effectiveness of interventions aimed at improving SP compliance. The data collected during this period will be invaluable for future strategic initiatives.

## Author Contributions

**Seyda Seren Intepeler:** conceptualization, project administration, funding acquisition, writing – original draft. **Menevse Yildirim:** conceptualization, data curation, funding acquisition, formal analysis, investigation, methodology, project administration, writing – original draft, visualization. **Simon Ching Lam:** writing – original draft, writing – review and editing, supervision.

## Funding

This study constitutes one of the stages of an extensive study conducted in a hospital to improve nurses' work environments and is supported by Dokuz Eylul University Department of Scientific Research Projects (Project number: 2018.KB.SAG.019). If the research is accepted for publication, our institution will cover the publication fee in line with the agreement between TUBITAK and Wiley.

## Ethics Statement

To conduct the research, institutional permissions were received from the Noninvasive Research Ethics Committee where the researchers worked (Date: 10.03.2016, Decision No: 2016107‐27), from the scale owner Simon Ching Lam, and from the Education and Research Hospital where the study was carried out. Informed consent was obtained from the nurses before the training. All the information provided by the nurses was kept confidential.

## Conflicts of Interest

The authors declare no conflicts of interest.

## Data Availability

The data that support the findings of this study are available from the corresponding author upon reasonable request.
